# IFI44 Promotes Clear Cell Renal Cell Carcinoma Progression via PRDX1 and Predicts Poor Prognosis

**DOI:** 10.34133/research.1102

**Published:** 2026-03-06

**Authors:** Yipeng Xu, Renjun Gu, Hao Zhang, Qiyin Zhou, Yongbo Wang, He Wang, Wei Zhu, Desheng Zhu, Mei Song, Junjie Bai, Jun Lin, Song Zheng, Jianhui Chen, Shaoxing Zhu

**Affiliations:** ^1^ Department of Urology, Zhejiang Cancer Hospital, Hangzhou, P.R. China.; ^2^ The Key Laboratory of Zhejiang Province for Aptamers and Theranostics, Hangzhou Institute of Medicine, Chinese Academy of Sciences, Hangzhou, P.R. China.; ^3^ School of Chinese Medicine, Nanjing University of Chinese Medicine, Nanjing, P.R. China.; ^4^ Department of Gastroenterology and Hepatology, Jinling Hospital, Medical School of Nanjing University, Nanjing, P.R. China.; ^5^ College of Pharmacy, Zhejiang University of Technology, Hangzhou, P.R. China.; ^6^ Cixi Biomedical Research Institute, Wenzhou Medical University, Wenzhou, P.R. China.; ^7^ The Second Clinical Medical College, Zhejiang Chinese Medical University, Hangzhou, P.R. China.; ^8^ Department of Urology, The Affiliated Hospital of Jiaxing University, Jiaxing, P.R. China.; ^9^ Department of Urology, Affiliated Jinhua Hospital, Zhejiang University School of Medicine, Jinhua, P.R. China.; ^10^ Department of Ultrasound, Zhejiang Cancer Hospital, Hangzhou, P.R. China.; ^11^ Shengli Clinical College of Fujian Medical University, Fuzhou, P.R. China.; ^12^ Department of Urology, Fuzhou University Affiliated Provincial Hospital, Fuzhou, P.R. China.; ^13^ The Graduate School of Fujian Medical University, Fuzhou, P.R. China.; ^14^ Department of Urology, Fujian Medical University Union Hospital, Fuzhou, P.R. China.

## Abstract

Clear cell renal cell carcinoma (ccRCC) is a lethal urologic malignancy with limited biomarkers for prognosis and therapeutic stratification. Interferon-induced protein 44 (IFI44) has been implicated in immune regulation, but its role in ccRCC is unclear. To address this gap, we comprehensively investigated the clinical significance, biological roles, and molecular mechanisms of IFI44 in ccRCC pathogenesis. Using integrative transcriptomic analysis of the Gene Expression Omnibus and the Cancer Genome Atlas-Kidney Clear Cell Carcinoma cohorts, we first identified IFI44 as a key candidate gene. Bioinformatic enrichment analyses and immune infiltration profiling were conducted to investigate potential mechanisms. In parallel, we established ccRCC cell lines with stable IFI44 knockdown and evaluated phenotypic changes using Cell Counting Kit-8, Transwell assays, wound-healing assays, and flow cytometry, thereby examining cell proliferation, apoptosis, migration, and invasion. We observed that IFI44 was markedly elevated in ccRCC tissues, and its increased level was closely associated with advanced tumor stage and poorer patient survival. Enrichment analyses indicated that IFI44 participates in pathways related to viral response, RNA splicing, and mRNA processing. Moreover, elevated IFI44 expression may be associated with an immunosuppressive tumor microenvironment, as suggested by increased infiltration of effector T cells and M1 macrophages, along with decreased infiltration of activated dendritic cells. In mechanistic studies, IFI44 knockdown markedly suppressed cell proliferation, triggered apoptosis, and reduced both migratory and invasive capacities, whereas PRDX1 overexpression rescued these phenotypes and PRDX1 was shown to interact with IFI44. In summary, the data show that IFI44 acts as an oncogene in ccRCC, promoting tumor progression through its interaction with PRDX1 while also shaping an immunosuppressive microenvironment, and suggest that IFI44 is a promising biomarker of prognosis and candidate therapeutic target for ccRCC.

## Introduction

Renal cell carcinoma (RCC) stems from renal tubular epithelial cells and represents ~90% of adult kidney tumors [1–3]. This disease is highly heterogeneous at histological and molecular levels [3]. Clear cell renal cell carcinoma (ccRCC) is the leading histological subtype of RCC, accounting for 70% to 80% of RCC diagnoses [4,5]. At the molecular level, ccRCC is distinguished by loss-of-function alterations in the von Hippel–Lindau tumor suppressor gene, leading to dysregulated hypoxia-inducible factor signaling that drives angiogenesis, metabolic reprogramming, and remodeling of the immune microenvironment [6,7]. Concomitant alterations in PBRM1, BAP1, SETD2 and related genes further exacerbate intratumoral heterogeneity and biological diversity [8]. These pathobiological features are tightly linked to tumor progression, underscoring the importance of identifying key molecular drivers to refine diagnostic and therapeutic strategies.

Clinical ccRCC management is challenging due to its strong metastatic potential. About 20% to 30% of cases have distant metastases at diagnosis, and another 23% develop recurrence or progression after curative surgery [9]. Metastases are often found in the lungs, bones, liver, and lymph nodes [10]. Once metastatic, RCC becomes more difficult to treat, with a dismal prognosis, with less than 15% of patients with advanced disease surviving for 5 years [11]. Before anti-angiogenic agents were introduced, cytokine therapy served as the mainstay treatment for patients with locally advanced or metastatic RCC [12]. Although a minority of patients achieved durable remission, cytokine therapy was associated with modest overall response rates and substantial toxicity [13]. Following 2005, therapies inhibiting the vascular endothelial growth factor receptor (VEGFR) and mammalian target of rapamycin pathways led to significant improvements in progression-free survival [14,15]. Despite initial benefit, most patients eventually experience acquired resistance to these therapies [16]. In recent years, immune checkpoint inhibitors (ICIs)—either used alongside other immunotherapies or combined with targeted agents—have become the standard first-line treatment, leading to improved response rates and overall survival (OS) benefits [17,18]. Nevertheless, pronounced heterogeneity in treatment response, high rates of immune-related adverse events, and scarcity of reliable predictive biomarkers continue to limit clinical decision-making [13]. Thus, deeper investigation into ccRCC molecular mechanisms and identification of novel prognostic and therapeutic targets remain imperative.

The interferon-induced protein 44 (IFI44) gene maps to chromosome 1p31.1 in humans [19]. It was initially identified in liver cells from hepatitis C virus-infected chimpanzees, and its human ortholog was subsequently confirmed to be an interferon-stimulated gene (ISG) induced by interferon-α/β [20]. Earlier research has demonstrated that the IFI44 gene is broadly involved in antiviral innate immune responses and various immune-related processes, and that it is commonly and significantly up-regulated in viral infections as well as autoimmune disorders, including systemic lupus erythematosus [21–24]. IFI44 exhibits heterogeneous functions across different solid tumors. In melanoma cells, IFI44 overexpression exerts an antiproliferative effect in vitro; in lung cancer, ectopic overexpression of IFI44 enhances tumor cell sensitivity to gefitinib; and in head and neck squamous cell carcinoma, IFI44/IFI44L act as downstream effectors of ACSL4, contributing to the promotion of tumor cell proliferation and invasion [25–27]. However, research on IFI44 in RCC is still in its early stages.

In this study, we systematically characterized IFI44’s expression, prognostic significance, functions, and mechanisms in ccRCC using integrated bioinformatics analyses, as well as in vitro cell experiments and in vivo animal experiments. Our findings support IFI44 as a candidate prognostic indicator and actionable target in ccRCC.

## Results

### IFI44 is significantly up-regulated in ccRCC and associated with poor prognosis

**Fig. 1.**
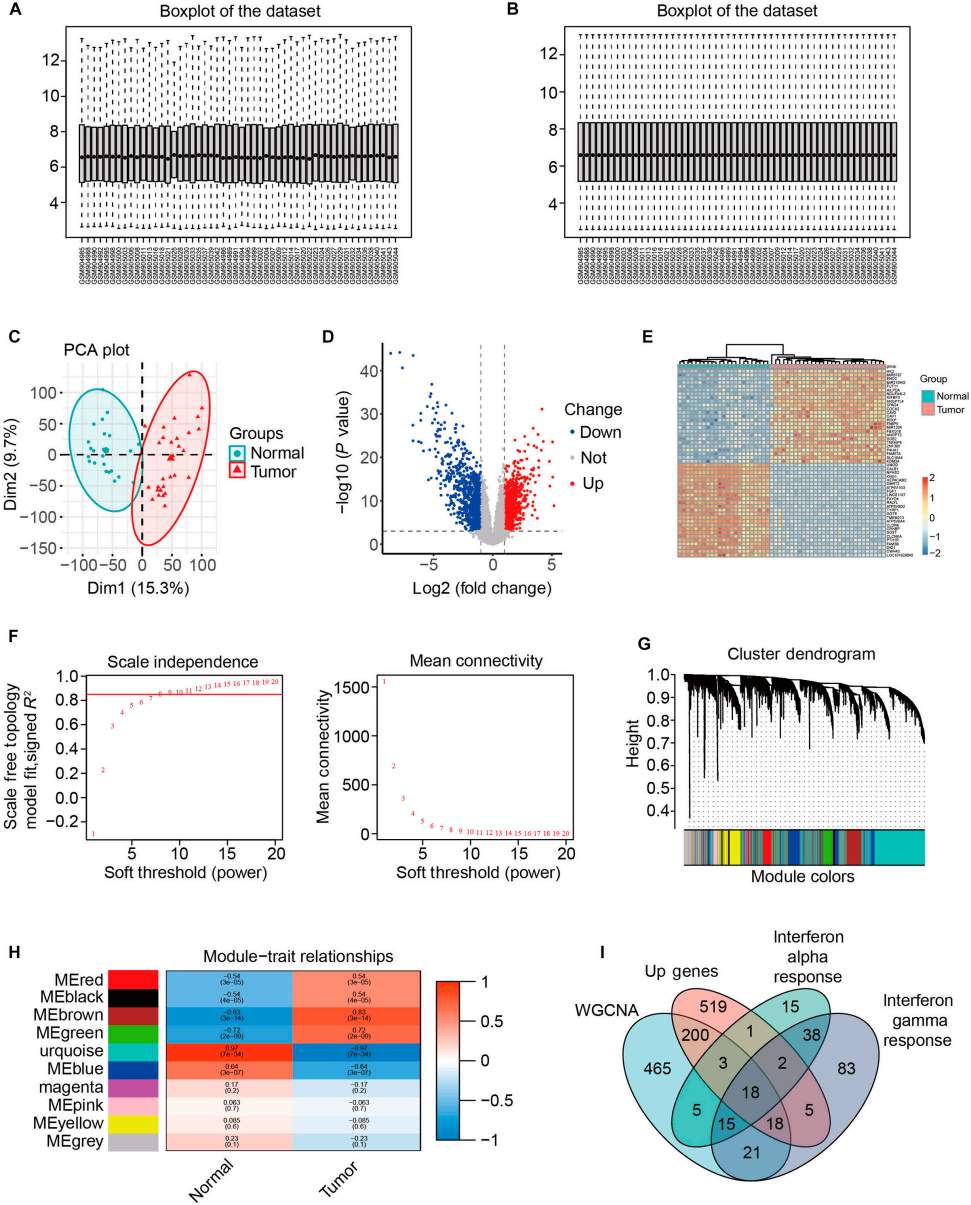
Module identification for clinical characteristics of clear cell renal cell carcinoma from data normalization and variance analysis. (A) The distribution of GSE36895 data before standardization, (B) The distribution of GSE36895 data after standardization. (C) The data distribution PCA after standardization. (D) Volcano plots show differential genes. (E) Heatmap shows differential genes. (F) Filter the WGCNA soft threshold. (G) Tree clustering of 5,000 highly variable genes was based on measured dissimilarity (1-TOM). The colored strip displays the output of the automated monolithic analysis. (H) Heatmap of module eigengene–trait relationships in ccRCC. We selected the MEbrown-grade block for subsequent analysis. (I) The Venn diagram shows 18 common genes, including 3 interferon family genes: IFI44, IFI44L, and IFI27.

**Fig. 2.**
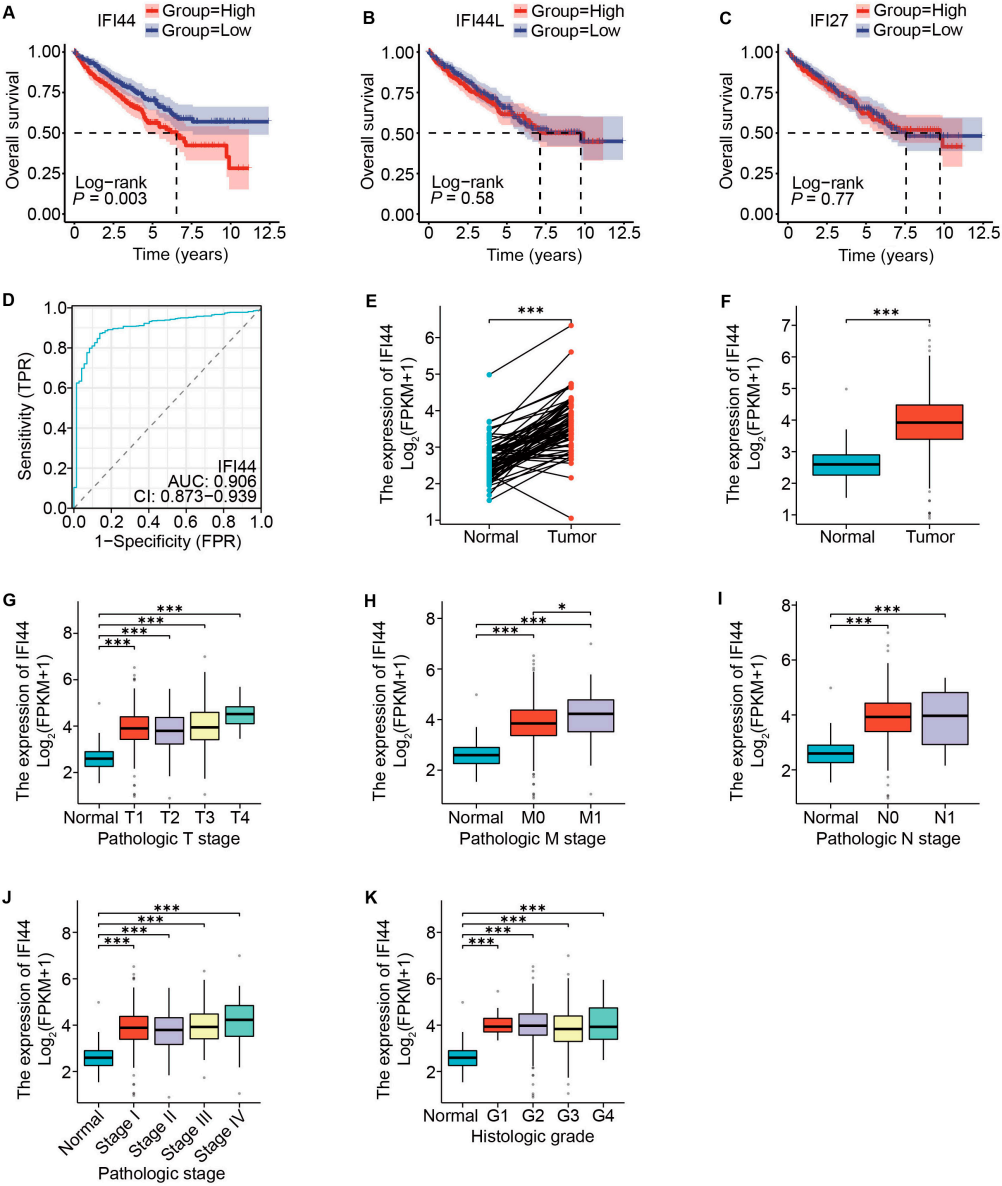
Selection of target genes and correlation of clinical features. (A to C) Kaplan–Meier survival analyses of IFI44, IFI44L, and IFI27; IFI44 was selected for subsequent analysis based on its prognostic significance. (D) ROC curve of the clinical diagnostic sensitivity of IFI44. (E and F) IFI44 expression in adjacent nontumor and tumor renal tissues (paired and unpaired comparisons). (G to K) Correlation between IFI44 expression and characteristics.

To systematically identify potential key driver genes and prognostic biomarkers in ccRCC, we analyzed the GSE36895 microarray dataset from the Gene Expression Omnibus (GEO) database. This dataset includes 29 tumor samples from ccRCC and 23 samples of adjacent nontumor kidney tissue. Following data normalization (Fig. 1A and B), PCA clearly distinguished tumor samples from nontumor tissues (Fig. 1C). Subsequently, differential expression was assessed using all 52 samples, and a volcano plot and hierarchical clustering heatmap were generated to visualize the differentially expressed genes (DEGs) (Fig. 1D and E). Using the weighted gene co-expression network analysis (WGCNA), we identified 745 genes belonging to the MEbrown module, which showed the highest correlation with tumor status (Fig. 1F to H). By intersecting these module genes with the DEGs, we identified 3 interferon-related family genes: IFI44, IFI44L, and IFI27 (Fig. 1I). Among them, only IFI44 demonstrated significant prognostic value in survival analysis and showed strong diagnostic performance (ROC AUC = 0.83) (95% CI: 0.76 to 0.90) (Fig. 2A to D). Therefore, IFI44 was selected as the principal subject of the present study. To investigate the association between IFI44 expression and clinicopathological characteristics, we analyzed IFI44 expression levels across different T, N, M stages, overall TNM stage, and histological grade using the TCGA-KIRC cohort. A significant elevation of IFI44 expression was observed in ccRCC tumor specimens relative to adjacent normal kidney specimens, and this up-regulation was statistically significant across overall TNM stage and grade subgroups (Fig. 2E to K).

### Functional enrichment and immune infiltration analysis

**Fig. 3.**
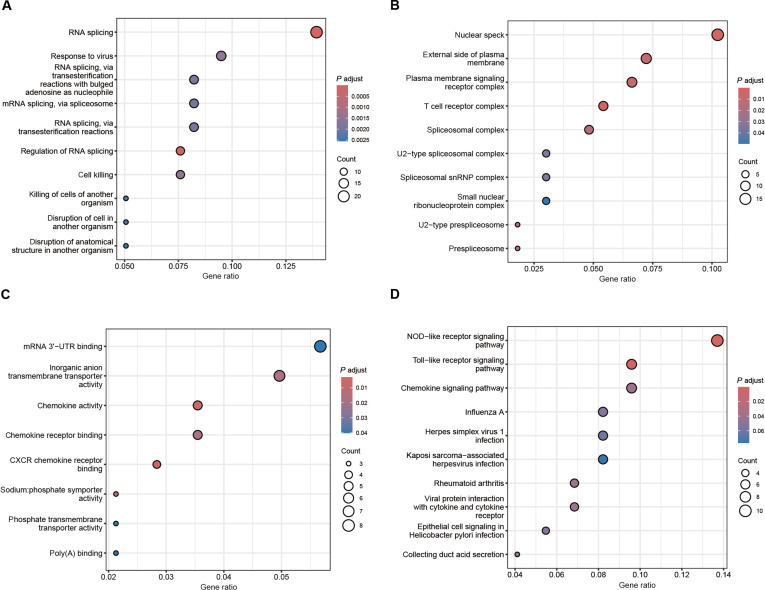
Functional enrichment profiling of IFI44-associated genes in ccRCC. (A) Gene Ontology (GO) analysis of biological processes (GO-BP). (B) GO analysis of cellular components (GO-CC). (C) GO analysis of molecular functions (GO-MF). (D) KEGG pathway enrichment analysis.

We interrogated DEGs comparing high- versus low-IFI44 ccRCC samples from the TCGA dataset through Gene Ontology (GO) and Kyoto Encyclopedia of Genes and Genomes (KEGG) enrichment (Fig. 3). These genes were primarily enriched in RNA splicing and related biological processes and immune and inflammation-related pathways such as “response to virus” and “cell killing” (Fig. 3A). Their encoded proteins were primarily localized to “nuclear speckles”, “plasma membrane signaling receptor complex”, “external side of the plasma membrane”, “T cell receptor complex”, and “spliceosomal complex” (Fig. 3B). Moreover, these genes were functionally enriched in “mRNA 3′-UTR binding”, “inorganic anion transmembrane transporter activity”, “chemokine receptor binding, including CXCR chemokine receptor binding”, and “chemokine activity” (Fig. 3C). KEGG pathway mapping enriched IFI44-related genes in NOD-like and Toll-like receptor pathways, chemokine pathway, multiple viral infection pathways (e.g., influenza A, herpes simplex virus, and Kaposi sarcoma-associated herpesvirus infections), and inflammatory disease pathways such as rheumatoid arthritis (Fig. 3D). Taken together, the data support the notion that in ccRCC, the IFI44 gene is closely involved in RNA splicing and posttranscriptional regulation processes, while also widely engaging in immune and inflammation signaling networks mediated by pattern recognition receptors and chemokines.

**Fig. 4.**
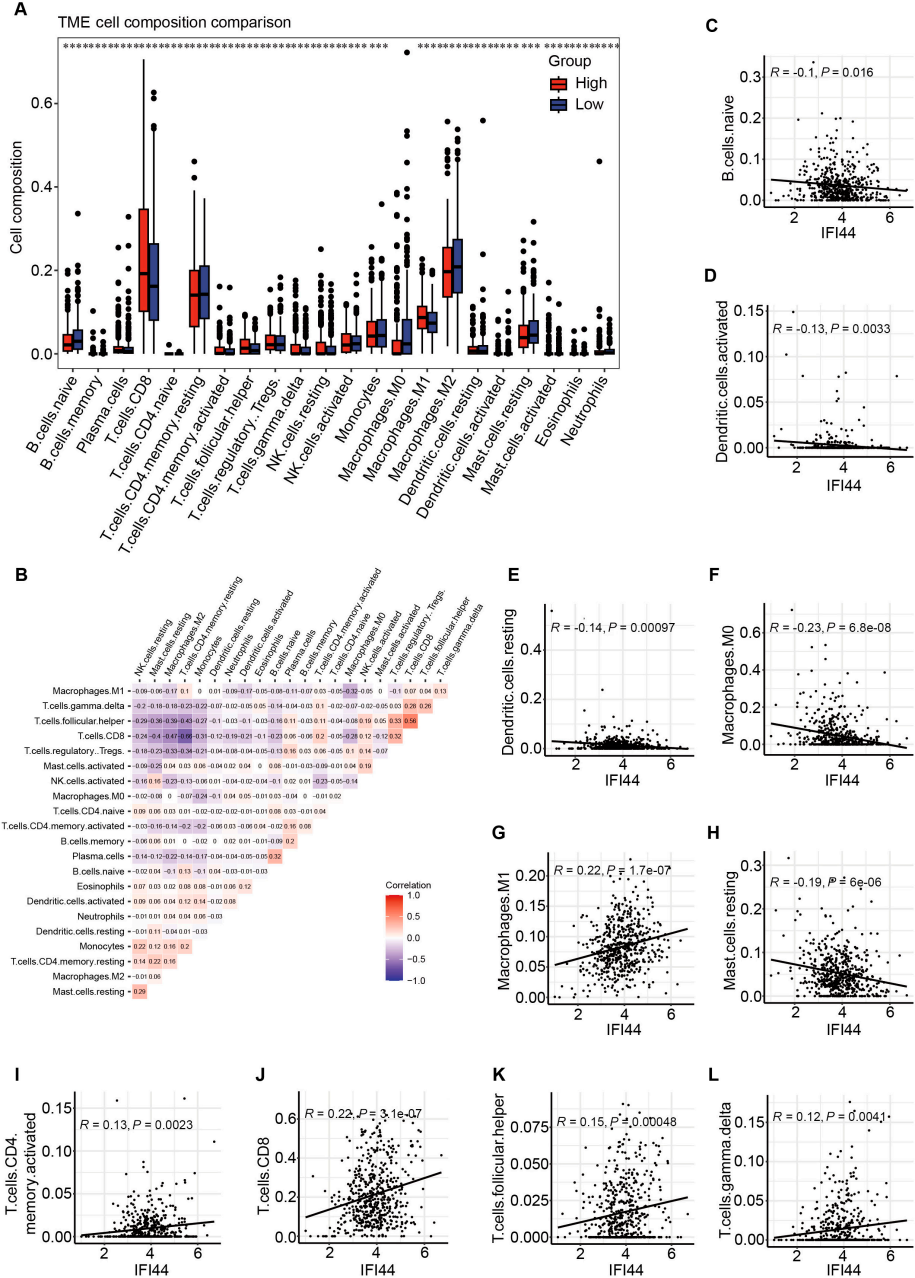
Immune cell infiltration analysis in 534 ccRCC samples from the TCGA cohort. (A) Immune landscape differences in tumors stratified by IFI44 expression (high vs. low). (B) Correlation matrix showing relationships among infiltrating immune cell populations. (C to L) Correlation of IFI44 expression with immune cell infiltration levels; only statistically significant correlation plots are shown.

To further evaluate the immune landscape, we assessed differences in immune cell infiltration between high- and low-IFI44 ccRCC samples (Fig. 4). The results show that samples with high IFI44 expression generally exhibit more active and immune-inflammatory-type infiltration. Among various immune cell types, the infiltration ratio was significantly higher in the high-expression group than in the low-expression group, especially in CD8 T cells, activated CD4 memory T cells, and others (Fig. 4A). The correlation matrix of immune cells further reveals a clear synergistic relationship between different infiltrating populations, with a highly positive correlation observed between M1 macrophages, activated CD4 memory T cells, CD8 T cells, and T gamma delta cells, among other effector immune cells (Fig. 4B). Furthermore, IFI44 expression correlated closely with the degree of infiltration across immune cell subsets (Fig. 4C to L), supporting a potential role for IFI44 in shaping the immune landscape of TME. Notably, despite higher overall immune cell infiltration in IFI44-high tumors, there is a declining trend in the infiltration of certain cells associated with antigen presentation or immune activation, such as activated dendritic cells. This pattern of enhanced immune activation coupled with impaired immune regulation suggests that high IFI44 expression may contribute to an imbalanced, partially immunosuppressive tumor microenvironment, thereby facilitating ccRCC progression and metastasis.

### IFI44 knockdown inhibits malignant phenotypes in RCC cells

**Fig. 5.**
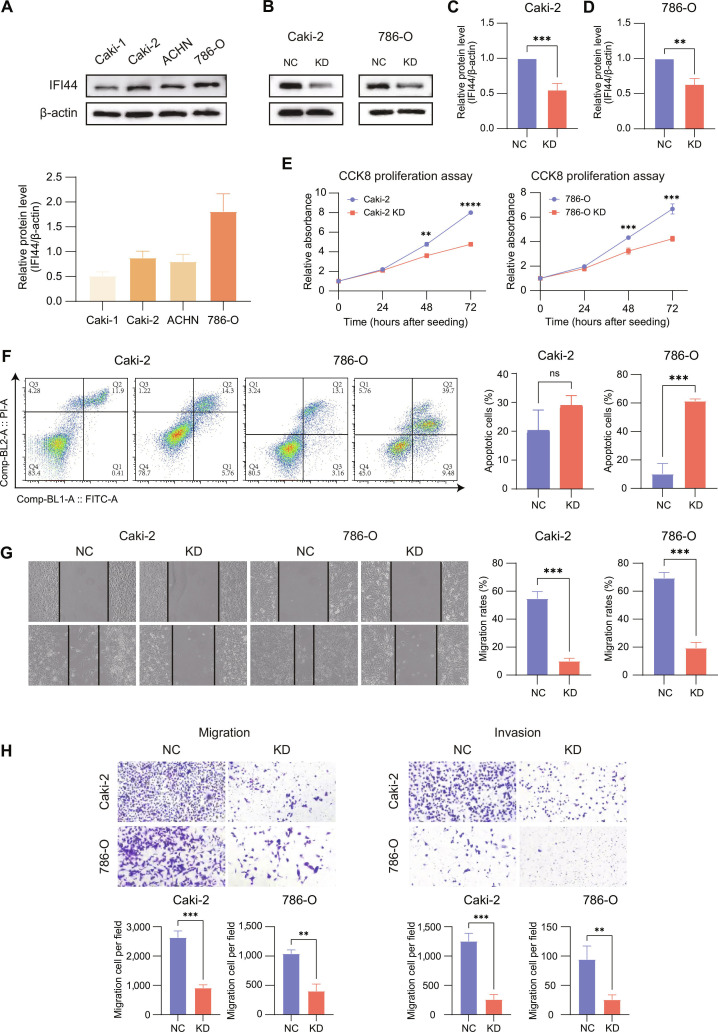
IFI44 knockdown inhibits malignant phenotypes in RCC cells. (A) IFI44 levels in RCC cell lines assessed by Western blot with β-actin as the control. (B) IFI44 protein levels assessed by Western blot in Caki-2 and 786-O cells following IFI44 knockdown (KD) or negative control (NC). (C and D) Densitometric quantification of IFI44 levels in Caki-2 (C) and 786-O (D) cells normalized to β-actin. (E) CCK-8 assay showing Caki-2 and 786-O cell proliferation at 0, 24, 48, and 72 h. (F) Flow cytometric analysis and quantification of Caki-2 and 786-O cell apoptosis following IFI44 KD. (G) Representative wound healing images and quantification of migration rates of Caki-2 and 786-O cells after KD or NC treatment. (H) Representative images of cell migration and invasion in Caki-2 and 786-O after KD or NC treatment, with corresponding quantification of migrated/invaded cells shown below.

To select appropriate cell lines for functional studies, we examined IFI44 protein expression across 4 RCC cell lines. Western blotting revealed relatively high IFI44 expression in Caki-2 and 786-O cells; therefore, these 2 lines were chosen for subsequent experiments (Fig. 5A). Stable IFI44 knockdown was achieved using lentiviral transduction (Fig. 5B to D). Cell counting kit-8 (CCK-8) assays revealed that IFI44 knockdown markedly attenuated proliferation in both cell lines (Fig. 5E). Flow cytometry revealed a significant increase in apoptosis following IFI44 silencing (Fig. 5F). Moreover, migratory and invasive capacities, as assessed by wound-healing and Transwell assays, were substantially diminished after the knockdown of IFI44 (Fig. 5G and H). Taken together, our data support a role for IFI44 in promoting renal cancer cell proliferation, whereas loss of IFI44 markedly reduces cell invasiveness.

### Repression of IFI44 inhibited the development of ccRCC in vivo

**Fig. 6.**
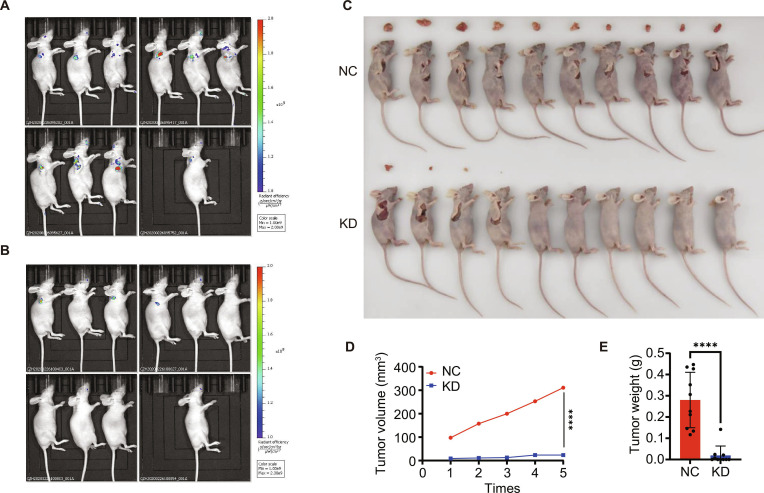
IFI44 promotes ccRCC progression and metastasis in vivo. (A) In vivo imaging of tumors in mice from the NC group. (B) In vivo imaging of tumors in mice from the IFI44 knockdown group. (C) Tumor size in mice of the NC group and the shIFI44 group. (D) Tumor growth curves comparison between the NC group and the shIFI44 group. (E) Tumor weight comparison between the NC group and the shIFI44 group.

To evaluate the tumor-promoting role of IFI44 in vivo, ACHN cells transduced with shIFI44 or NC were implanted into BALB/c nude mice. Specifically, these cells were injected into the right axillary region of nude mice, and once tumors were established, tumor dimensions were recorded at 3-day intervals until day 69. To effectively measure tumor size, metastasis, and severity, luciferase imaging was performed as depicted in Fig. 6A and B. Our findings showed that bioluminescence signal intensity was substantially greater in the NC group than in the shIFI44 group. Furthermore, tumor weight and tumor volume in the shIFI44 group were considerably smaller than those in the NC group, as evidenced in Fig. 6C to E. Importantly, mice in the shIFI44 group exhibited a noticeable reduction in the degree of metastasis relative to those in the NC group. In sum, these data demonstrate that IFI44 silencing effectively suppresses ccRCC growth and metastasis in vivo.

### PRDX1 was one of the targets of IFI44

**Fig. 7.**
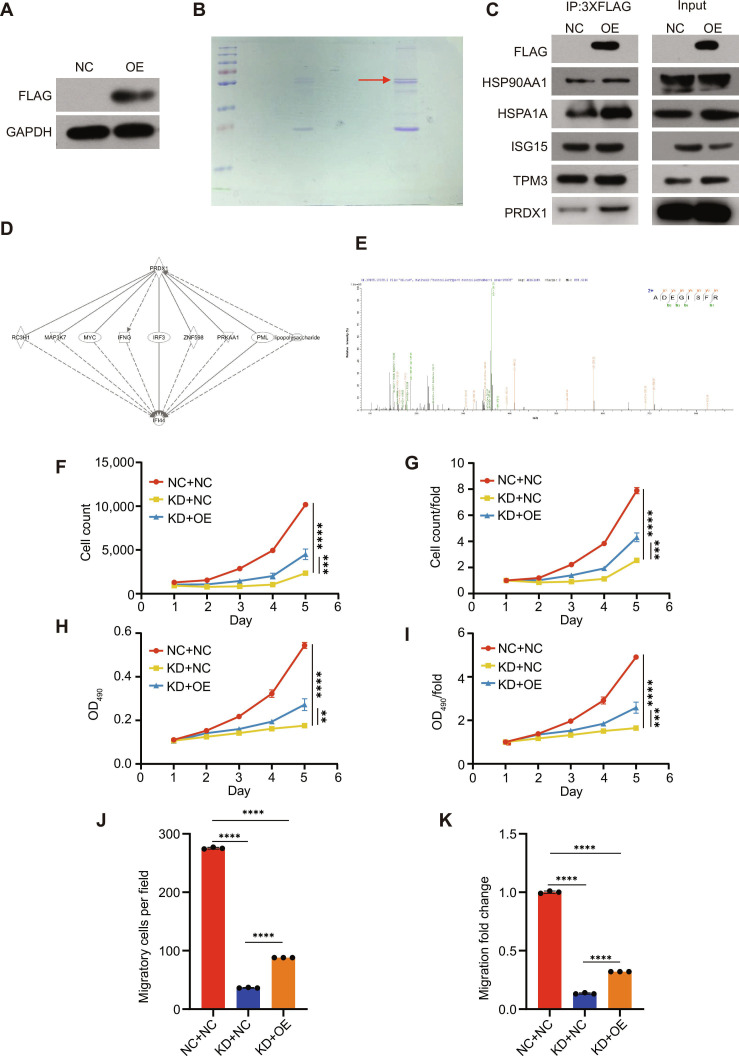
Co-IP identification of PRDX1 as an IFI44-interacting protein and its functional impact on cell proliferation and metastasis. (A) Western blot verifying successful establishment of stable ACHN cell lines expressing 3× FLAG-tagged IFI44. (B) SDS-PAGE analysis of immunoprecipitated IFI44 and associated proteins from IFI44-overexpressing and control cells. (C) Co-IP assays showing the interaction between IFI44 and PRDX1. PRDX1 was detected in both NC and IFI44-overexpression (OE) groups; however, PRDX1 enrichment in the IP fraction was markedly higher in the OE group, indicating a specific interaction with IFI44. (D) Co-IP reciprocal network analysis. (E) Co-IP–MS; some of the peaks observed in the figure include dehydration (−H_2_O) and deamination (−NH_3_) reactions. (F and G) Cell proliferation assays showing time-dependent changes in cell number among different treatment groups. (H and I) Quantification of proliferative activity differences among groups. (J and K) Migration and invasion assays showing changes in metastatic capacity and corresponding quantification. NC+NC, control cells infected with negative control viruses; KD+NC, IFI44 knockdown cells infected with negative control virus; KD+OE, IFI44 knockdown cells with PRDX1 overexpression.

To identify IFI44-interacting proteins, we employed co-immunoprecipitation in combination with mass spectrometry (Co-IP/MS) using ACHN cells overexpressing IFI44. A lentiviral construct encoding full-length IFI44 fused to an N-terminal 3× FLAG tag was generated and delivered into ACHN cells. An empty lentiviral vector served as the control (Fig. 7A). Cell lysates were incubated with the anti-3×FLAG beads to immunoprecipitate IFI44 and associated proteins. Immunoprecipitates from IFI44-overexpressing and control cells were resolved by sodium dodecyl sulfate-polyacrylamide gel electrophoresis (SDS-PAGE) (Fig. 7B). The selected gel lane was trypsin-digested and analyzed by liquid chromatography-tandem mass spectrometry (LC-MS/MS). In ACHN cells overexpressing IFI44, 331 peptides were identified, whereas only 147 peptides were detected in control cells. A total of 67 proteins showed relatively higher abundance in IFI44-overexpressing cells. The interaction between IFI44 and five candidate proteins was examined by Co-IP, which revealed a specific interaction between IFI44 and PRDX1 (Fig. 7C); this interaction was also confirmed by MS (Fig. 7D and E). However, the other 4 candidate IFI44-interacting proteins (HSP90AA1, HSPA1A, ISG15, and TPM3) were not confirmed. According to the results above, we speculated that IFI44 might regulate PRDX1 to facilitate renal cancer cell growth.

### PRDX1 rescued the impaired proliferation and migration caused by IFI44 knockdown

To investigate whether IFI44 promotes proliferation and migration in ccRCC cells through PRDX1, we constructed ACHN cells with IFI44 knockdown and simultaneous PRDX1 overexpression. As illustrated in Fig. 7F to I, overexpression of PRDX1 significantly attenuated the IFI44 knockdown-mediated suppression of proliferation. Specifically, cell proliferation rates were significantly reduced after IFI44 knockdown, but up-regulation of PRDX1 restored the proliferation rate, based on the MTT assay and cell-counting experiments. Moreover, up-regulation of PRDX1 also stimulated the migratory and invasive behaviors in ACHN cells despite the knockdown of IFI44 (Fig. 7J and K). These results indicate that PRDX1 functions downstream of IFI44 and can rescue proliferative and migratory defects induced by IFI44 depletion in ccRCC cells.

## Discussion

This research systematically characterized IFI44’s expression, functions, and molecular mechanisms in ccRCC using integrated bioinformatics analyses, as well as in vitro cell experiments and in vivo animal experiments. Integrated bioinformatic analyses showed that, compared with paracancerous renal tissues, IFI44 was markedly up-regulated in ccRCC tissues, and elevated IFI44 levels was closely associated with higher M stage and poor patient prognosis. On this basis, we further found that high IFI44 expression can significantly enhance proliferative capacity, migration, and invasiveness in ccRCC cells. In melanoma cells, IFI44 overexpression exhibits an antiproliferative effect in vitro; in non-small cell lung cancer, ectopic expression of IFI44 increases the sensitivity of tumor cells to gefitinib; and in head and neck squamous cell carcinoma, IFI44/IFI44L, as downstream effector molecules of ACSL4, participate in driving tumor cell growth and invasion [25–27]. Compared with previous studies, our study further confirms that IFI44 exhibits marked tissue specificity and functional heterogeneity across different tumor types, and suggests that IFI44 may serve as a promising prognostic biomarker and potential therapeutic target for ccRCC. It should be noted that the utility of IFI44 as an independent diagnostic biomarker still requires dedicated evaluation in diagnostic cohorts. It is worth further exploring that the functional differences of IFI44 among different tumor types may be shaped by the tumor-specific molecular background in which it resides. Most types of solid tumors carry multiple, or even dozens of, mutations in cancer genes, which determine the characteristics of the cancer [28,29]. Accordingly, the functional diversity of IFI44 is likely influenced by tumor-specific genetic backgrounds, co-regulatory networks, and microenvironmental contexts. In ccRCC, which is characterized by von Hippel–Lindau inactivation, metabolic reprogramming, and a unique immune microenvironment, IFI44 may be integrated into oncogenic regulatory networks driven by these features. This integration may shift IFI44 from a conventional antiviral or stress-response protein to a factor with pro-tumorigenic functions [30–32].

Findings from functional enrichment analyses indicated that the differentially expressed genes identified by stratifying samples according to IFI44 expression levels were significantly enriched in terms such as “nuclear speckle”, “spliceosomal complex”, and “mRNA 3′-UTR binding”. Among them, nuclear speckles were initially regarded as regions for the storage and modification of splicing factors, enriched with numerous RNA-binding proteins, splicing factors, and 3′-end processing factors, and were later proposed by researchers to potentially serve as dynamic hubs for the regulation of nuclear gene expression [33,34]. The above results suggest that the IFI44-related network may be closely associated with RNA splicing and mRNA processing. Taken together with previous studies indicating that aberrant alternative splicing is one of the important molecular characteristics of ccRCC and is tightly linked to tumor progression, prognosis, and remodeling of the tumor immune microenvironment [35,36], we speculate that IFI44 may promote the progression of ccRCC by regulating RNA splicing and mRNA processing. Secondly, the differentially expressed genes are significantly enriched in the NOD-like receptor, Toll-like receptor, and chemokine signaling pathways, and are closely related to various viral infection and inflammatory disease pathways, such as rheumatoid arthritis. These pathways are critical for the activation of type I interferon responses [37]. Consistent with our observations, prior studies have reported that IFI44 expression is up-regulated in inflammatory contexts [24,38,39]. However, because samples from inflammatory diseases were not included in our study, we were unable to directly evaluate whether IFI44 exhibits an intermediate expression pattern between normal and tumor tissues. Therefore, future studies should include renal inflammatory diseases or other inflammatory disease samples for systematic comparisons to further define its expression profile across different pathological conditions. Experimental studies have shown that IFI44 can interact with molecules such as FKBP5 to regulate the activation of the IKK family and downstream IRF3/NF-κB, thereby negatively regulating the type I and type III interferon pathways [22]. Therefore, IFI44-mediated modulation of these signaling pathways may affect the functional state of immune cells, thereby influencing the tumor immune escape mechanism. The immune microenvironment analysis further supports this view. Tumors in the high IFI44 expression group exhibit enhanced immune cell infiltration, particularly in M1 macrophages and effector T cells. This suggests that high expression of IFI44 could drive immune cell recruitment and activation, thus improving the immune response. However, despite the increased immune cell infiltration, the infiltration of activated dendritic cells showed a decreasing trend, indicating that the immune response may not have been effectively initiated or could be suppressed in certain aspects. The dysfunction of dendritic cells, which are important antigen-presenting cells, may affect tumor immune surveillance and potentially provide an opportunity for tumor cells to evade immune detection [40]. This phenomenon reveals a complex immune microenvironment characterized by the coexistence of “immune activation and impaired immune regulation”, and this imbalance may facilitate immune evasion and metastatic progression. It may also affect the tumor’s therapeutic response to ICI treatment. Translational studies further indicate that patients with a T-effector-high/angiogenesis-low gene expression signature experience the greatest clinical benefit from ICI combination therapy [41]. Therefore, a deeper understanding of this immune imbalance not only clarifies differential tumor responses to ICI therapy but also offers a theoretical framework for optimizing immunotherapy strategies.

**Fig. 8.**
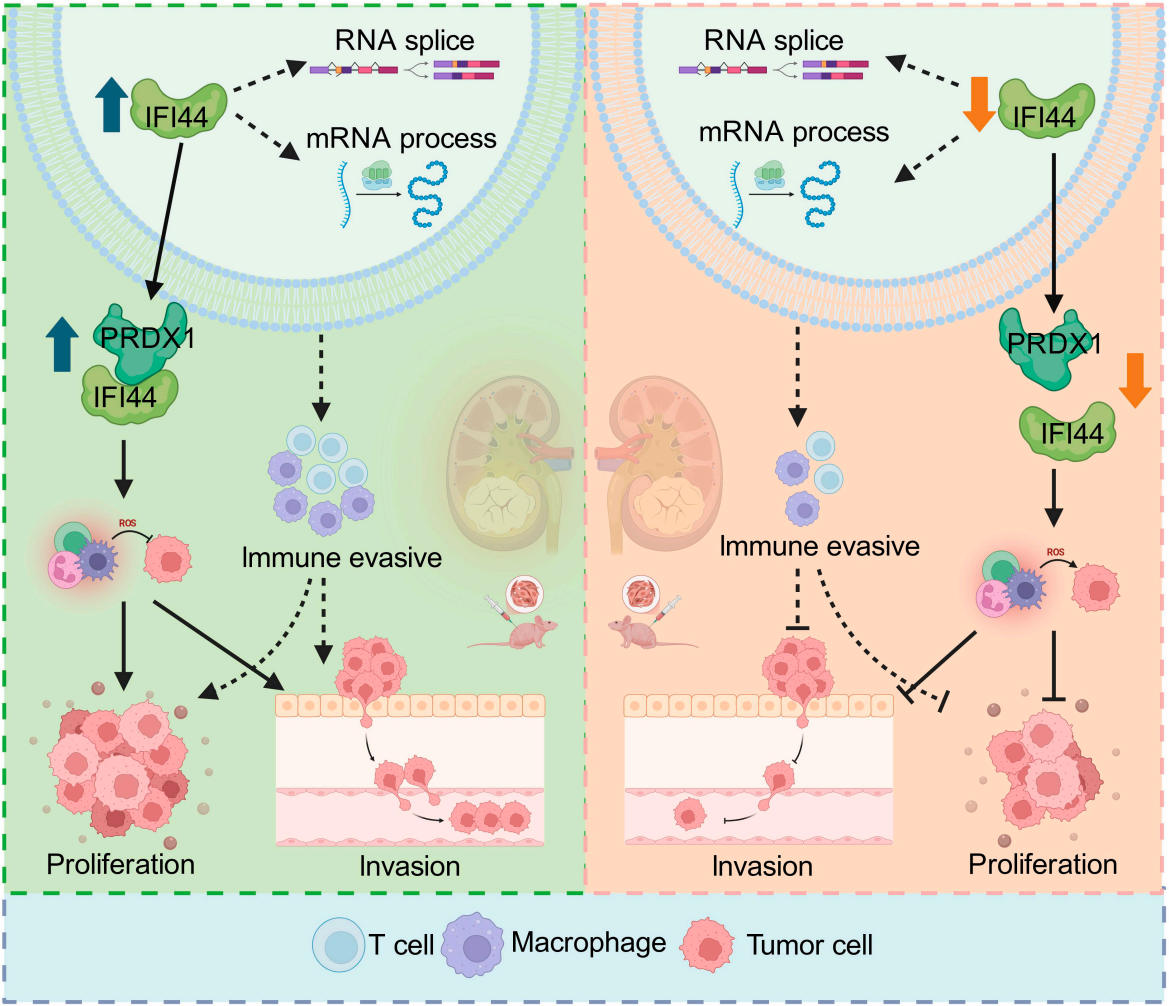
The molecular mechanism by which IFI44 promotes the progression of ccRCC. After high expression of IFI44 in ccRCC cells, it forms a complex with PRDX1, enhancing its antioxidant capacity and signal regulatory function, allowing the cells to maintain a higher survival rate and migratory ability under oxidative stress and metabolic overload conditions. Meanwhile, IFI44, through its role in RNA splicing and mRNA processing, regulates the abnormal expression of related genes and reshapes the immune microenvironment, thereby promoting tumor progression and immune escape (created with BioRender).

In vitro experiments further confirmed the pro-tumorigenic role of IFI44. Suppression of IFI44 markedly inhibited renal cancer cell proliferation and induced apoptosis, whereas high IFI44 expression enhanced cell invasion and metastatic potential, thereby accelerating tumor progression. In vivo experiments demonstrated consistent results, providing additional evidence that IFI44 promotes ccRCC tumorigenesis and development. Mechanistically, we identified PRDX1 as a key interacting partner of IFI44. Through protein–protein interaction analyses and functional rescue experiments, we identified the IFI44–PRDX1 axis as a key driver of malignant phenotype in ccRCC. PRDX1 belongs to the peroxiredoxin family and is an important molecule in regulating intracellular ROS levels and redox homeostasis. It not only scavenges hydrogen peroxide but also acts as a “redox signaling transducer” to regulate multiple pathways, including MAPK and NF-κB [42,43]. Accumulating evidence indicates that PRDX1 is up-regulated in multiple malignancies, including breast and lung cancers, and has been implicated in promoting tumor cell proliferation and migration, inhibiting apoptosis, and conferring chemotherapy resistance [43]. Our Co-IP–MS results show that in IFI44-overexpressing ACHN cells, PRDX1 was the only interacting protein that was identified by MS and successfully validated by Co-IP, whereas other candidate molecules (e.g., HSP90AA1, HSPA1A, ISG15, and TPM3) failed to be confirmed in the dual validation. This selective interaction indicates a strong specific association between IFI44 and PRDX1, rather than a general “sticky effect”. More compellingly, functional rescue experiments showed that PRDX1 overexpression restored proliferation and migration in IFI44-depleted cells, establishing PRDX1 as a critical downstream effector of IFI44. Based on the known functions of PRDX1 in ROS regulation and signal transduction, a more complete mechanistic hypothesis can be proposed: After high expression of IFI44 in ccRCC cells, it forms a complex with PRDX1, enhancing its antioxidant capacity and signal regulatory function, allowing the cells to maintain a higher survival rate and migratory ability under oxidative stress and metabolic overload conditions. Meanwhile, IFI44, through its role in RNA splicing and mRNA processing, regulates the abnormal expression of related genes and reshapes the immune microenvironment, thereby promoting tumor progression and immune escape (Fig. 8).

For early-stage ccRCC, surgical intervention is the standard clinical approach [44]. Although immunotherapy is more likely than VEGFR-targeted therapy to achieve durable responses in advanced RCC, and clinical trials have shown that pembrolizumab can improve disease-free survival (DFS) in patients at high risk of recurrence, OS data remain immature, biomarkers to select patients and avoid overtreatment are lacking, and other ongoing adjuvant ICI trials either have not yet reported results or have yielded negative DFS outcomes; consequently, current guidelines provide only a weak recommendation for adjuvant pembrolizumab [45]. However, in-depth analyses of the 3 negative trials indicate that the benefit of immunotherapy is largely influenced by trial design, patient heterogeneity, and the specific interventions employed. These findings also highlight that current prognostic indicators are insufficient to effectively guide the selection of patients for immunotherapy, underscoring an urgent need for more precise biomarkers to inform clinical decision-making and future research directions [45].

In this clinical context, the evidence revealed by this study shows that elevated IFI44 expression correlates strongly with tumor progression, unfavorable prognosis, and an immunosuppressive microenvironment, suggesting that IFI44 may become a promising molecular target for both prognosis assessment and therapeutic intervention in ccRCC. It is necessary to further validate the predictive value of IFI44 in prospective clinical cohorts and in populations receiving ICI treatment, explore an IFI44-based risk stratification model, and investigate the impact of IFI44-targeted interventions on the efficacy and resistance patterns of immunotherapy. This would provide a more solid biological basis for optimizing systemic treatment strategies for ccRCC and achieving truly personalized immunotherapy. In future clinical translation, depending on the intended application scenario, both enzyme-linked immunosorbent assay and fluorescence-based detection of IFI44 may be feasible; however, this study did not establish or validate dedicated assays in either format, and further research is required to develop clinically applicable detection methods.

In summary, our findings show that IFI44 serves as an important oncogenic factor in ccRCC, accelerating tumor progression by enhancing cell proliferation, migration, invasion, and shaping an immunosuppressive tumor microenvironment. IFI44 may represent a potential prognostic marker and treatment target, offering new perspectives for the development of precision medicine for this refractory malignancy.

## Materials and Methods

### Acquisition of RCC gene expression data

Gene expression data and associated clinical information for the GSE36895 dataset were from the GEO (https://www.ncbi.nlm.nih.gov/geo/). This dataset consists of 29 human ccRCC tumor tissues and 23 adjacent nontumor kidney tissues. The 24 mouse samples included in the original dataset were excluded from subsequent analyses.

### Differential analysis and target gene identification

Raw expression data were standardized using the limma R package, and normalization results were assessed with the FactoMineR package. DEGs between tumor and adjacent nontumor tissues were assessed using limma according to average expression value (AveExpr) > 5, |log fold change| > 1, and *P* < 0.001. WGCNA was applied to the 5,000 most variable DEGs to detect ccRCC-associated modules, applying a soft-thresholding power of 8 for scale-free topology. A topological overlap matrix was constructed, and module detection was performed based on hierarchical clustering. Interferon-related genes were downloaded from the hallmark gene set collection (h.all.v2025.1.Hs.symbols.gmt) in MSigDB (www.gsea-msigdb.org). Two interferon-related pathways were extracted. The intersection of DEGs and genes within the tumor-associated WGCNA module was identified using the VennDiagram R package to screen interferon-stimulated genes relevant to ccRCC.

### TCGA data processing and clinical analysis

TCGA ccRCC RNA-seq and clinical data were from the UCSC Xena browser (http://xena.ucsc.edu/), including HTSeq-FPKM-normalized expression data, survival information, and the gene annotation file gencode.v22.annotation.gene.probeMap. Expression matrices were re-annotated based on probe-to-gene mappings and merged with clinical data. After quality control, the cohort comprised 535 ccRCC tumor samples and 72 adjacent normal tissues. Gene expression differences were plotted using the ggplot2 R package, and Kaplan–Meier curves were produced using survminer.

### Functional enrichment and immune infiltration analysis

DEGs were screened using the R package “limma”. Functional enrichment was assessed based on KEGG, and GO. Immune cell composition across sample groups was assessed using the “CIBERSORT” algorithm approach to estimate the relative fractions of 22 immune cell categories based on bulk gene expression profiles.

### Cultivation of renal cell lines

The human RCC cell lines 786-O (TCHu186), Caki-1 (TCHu135), Caki-2 (TCHu251), and ACHN (TCHu199) were sourced from the American Type Culture Collection in Shanghai, China. Cells were routinely cultured in RPMI-1640 medium supplemented with 10% fetal bovine serum (FBS; Gibco, New York, USA) and kept at 37 °C in a 5% CO₂ humidified incubator.

### Plasmid construction and cell transfection

Lentiviral particles were produced by transfecting 293T cells with shIFI44 plasmids using Lipofectamine 2000 (11668030, Thermo Fisher Scientific, USA). Viral supernatants were collected 48 h posttransfection and subsequently concentrated. RCC cells were infected with the purified virus and selected with puromycin for 48 h. After 3 passages, knockdown/overexpression efficiency was confirmed by Western blotting.

### Western blotting

Total cellular proteins were isolated using radio immunoprecipitation assay buffer with protease and phosphatase inhibitors and quantified using a bicinchoninic acid assay. Proteins were resolved by SDS-PAGE and transferred onto polyvinylidene difluoride (PVDF) membranes. Following blocking with 5% nonfat milk in Tris-buffered saline with Tween-20 (TBST) for 1 h at room temperature, membranes were soaked in primary antibody solution overnight at 4 °C, rinsed with TBST, and reacted with horseradish peroxidase-conjugated secondary antibodies. Bands were presented using enhanced chemiluminescence reagents.

### Cell proliferation assay

The CCK-8 assay served to measure cell proliferation. Cells were placed in 96-well plates at 3,000 cells per well. After overnight attachment, cells were incubated with 10 μl of CCK-8 solution at 24, 48, and 72 h. Absorbance readings at 450 nm were taken, and growth curves were plotted.

### Cell apoptosis analysis

Cells, ranging from 1 to 5 × 10^5^, were washed with phosphate-buffered saline (PBS), resuspended in 100 μl of binding buffer, and then incubated with 5 μl of Annexin V–fluorescein isothiocyanate and 5 μl of propidium iodide. The samples were incubated in darkness for 10 to 15 min before being analyzed by flow cytometry, with data processed through FlowJo software.

### In vitro wound healing assay

Cells were grown until they reached 80% to 90% confluence, then scratched with a sterile pipette tip and rinsed with PBS. Cells were then cultured in serum-free medium at 37 °C, 5% CO₂. Images were captured at 0 and 24 h, and migration was quantified using ImageJ.

### Transwell assay

In the migration assay, cells were trypsinized and resuspended in a medium without serum, then 100 μl of the cell suspension, containing 10,000 cells, was placed in the upper chamber. A complete medium containing 10% FBS was introduced to the lower chamber, reaching a total volume of 500 μl. The chambers were then incubated for 48 h at 37 °C with 5% CO₂. In the invasion assay, Matrigel was used to precoat the chambers. A cotton swab was carefully used to remove nonmigrated cells. The membranes were fixed with 4% paraformaldehyde for 15 min, followed by staining with crystal violet for 20 min. The membranes were washed with water and air-dried, and 5 random fields were photographed under a microscope. Migrated cells were manually counted.

### Co-IP–MS analysis

After harvesting the cells, they were washed twice with PBS. Cells were lysed for 15 min at 4 ℃, and clarified by spinning. IFI44–3× FLAG complexes were immunoprecipitated with anti-FLAG magnetic beads (Sigma), with empty vector lysates as controls. Immunoprecipitants were resolved by SDS-PAGE, excised, and subjected to MS. The excised gel bands were subjected to in-gel digestion, followed by LC-MS/MS analysis on a Q Exactive Plus mass spectrometer coupled to an Easy nLC system. Raw data were searched against the database using ProteomeDiscoverer/MASCOT. Proteins were identified against the UniProt database, with UniquePeptides count and coverage percentage used as auxiliary criteria. 

### In vivo experiments

Animal studies were ratified by the Animal Ethical Committee of the Hangzhou Institute of Medicine, Chinese Academy of Sciences. Four-week-old female BALB/c nude mice (Beijing Vitonlihua Laboratory Animal Technology Co., Ltd.) were randomly grouped (*n* = 10 per group). ACHN cells (1 × 10^7^) were injected subcutaneously into the mouse’s right armpit. Tumor volume was monitored every 3 days starting on day 57 after injection. Tumor metastasis in vivo was monitored using an in vivo imaging system. At 69 days postinjection, mice were sacrificed and the tumors were excised and weighed.

### Statistical analysis

All experiments were repeated 3 times independently, and results were reported as mean ± SD. Quantitative data were assessed using Student’s *t* test or one-way analysis of variance, with *P* < 0.05 defined as statistically significant. Analyses were performed in GraphPad Prism 5 (GraphPad, USA). Significance is indicated as ns (*P* > 0.5), *P < 0.05, **P < 0.01, ***P < 0.001, and ****P < 0.0001.

## Ethical Approval

Animal experiments in this study were approved by the Institutional Animal Care and Use Committee of the Hangzhou Institute of Medicine, Chinese Academy of Sciences (Approval Number: AP2024-10-0228).

## Data Availability

The data that support the findings of this study are available from the corresponding authors upon reasonable request.
