# Supplementary material for: IFI44 Promotes Clear Cell Renal Cell Carcinoma Progression via PRDX1 and Predicts Poor Prognosis
Source: Research (Wash D C). 2026 Mar 6;9:1102. doi: 10.34133/research.1102 (PMC12963643; doi:10.34133/research.1102)
Supplement: Supplementary 1 — Data File S1 [file research.1102.f1.docx]

**Front Matter**

Title

IFI44 Promotes Clear Cell Renal Cell Carcinoma Progression via PRDX1 and Predicts Poor Prognosis

**Authors**

Yipeng Xu^1, 2†^, Renjun Gu^3, 4†^, Hao Zhang^5†^, Qiyin Zhou^1^, Yongbo Wang^6^, He Wang^7^, Wei Zhu^8^, Desheng Zhu^9^, Mei Song^10^, Junjie Bai^11, 12, 13^, Jun Lin^14^, Song Zheng^14*^, Jianhui Chen^14*^, Shaoxing Zhu^14*^

**Affiliations**

^1^Department of Urology, Zhejiang Cancer hospital, Hangzhou, P.R. China.

^2^The Key Laboratory of Zhejiang Province for Aptamers and Theranostics, Hangzhou Institute of Medicine, Chinese Academy of Sciences, Hangzhou, P.R. China.

^3^School of Chinese Medicine, Nanjing University of Chinese Medicine, Nanjing, P.R. China.

^4^Department of Gastroenterology and Hepatology, Jinling Hospital, Medical School of Nanjing University, Nanjing, P.R. China.

^5^College of pharmacy, Zhejiang University of Technology, Hangzhou, P.R. China.

^6^Cixi Biomedical Research Institute, Wenzhou Medical University, Wenzhou, P.R. China.

^7^The Second Clinical Medical College, Zhejiang Chinese Medical University, Hangzhou, P.R. China.

^8^Department of Urology, The Affiliated Hospital of Jiaxing University, Jiaxing, P.R. China.

^9^Department of Urology, Affiliated Jinhua Hospital, Zhejiang University School of Medicine, Jinhua, P.R. China.

^10^Department of Ultrasound, Zhejiang Cancer hospital, Hangzhou, P.R. China.

^11^Shengli Clinical College of Fujian Medical University, Fuzhou, P.R. China.

^12^Department of Urology, Fuzhou University Affiliated Provincial Hospital, Fuzhou, P.R. China.

^13^The Graduate School of Fujian Medical University, Fuzhou, P.R. China.

^14^Department of Urology, Fujian Medical University Union Hospital, Fuzhou, P.R. China.

^*^Address correspondence to: **Shaoxing Zhu;** [**zsxing2005@126.com**](mailto:zsxing2005@126.com;) **; Jianhui Chen;** [**chenjianhui1983@qq.com**](mailto:chenjianhui1983@qq.com)**; and** Song Zheng; [zhengwu_99@aliyun.com](mailto:zhengwu_99@aliyun.com;)

^†^These authors contributed equally to this work.

**SUPPLEMENTARY MATERIALS**

**Cell proliferation assay**

Cell proliferation was assessed by Celigo-based cell counting. Cells were seeded into 96-well plates at 1,000 cells per well in triplicate and cultured at 37°C in a humidified incubator with 5% CO₂. Proliferation was monitored using a Celigo Image Cytometer for 5 consecutive days.

**MTT assay**

Cells in the logarithmic growth phase were trypsinized, resuspended in complete medium, and counted. Cells were seeded into 96-well plates at 2,000 cells per well in triplicate. Beginning the day after seeding, 20 μL of MTT solution (5 mg/mL) was added to each well 4 h before daily measurement, without changing the medium. After 4 h of incubation, the medium was carefully removed without disturbing the formazan crystals, and 100 μL of DMSO was added to dissolve the formazan. Plates were shaken for 2–5 min, and absorbance was measured at 490 nm using a microplate reader. Measurements were performed daily for 5 consecutive days, and growth curves were generated accordingly.
